# Efficient and Simple Production of Insulin-Producing Cells from Embryonal Carcinoma Stem Cells Using Mouse Neonate Pancreas Extract, As a Natural Inducer

**DOI:** 10.1371/journal.pone.0090885

**Published:** 2014-03-10

**Authors:** Marzieh Ebrahimie, Fariba Esmaeili, Somayeh Cheraghi, Fariba Houshmand, Leila Shabani, Esmaeil Ebrahimie

**Affiliations:** 1 Department of Biology, Faculty of Basic Sciences, Shahrekord University, Shahrekord, Iran; 2 Department of Biology, Faculty of Basic Sciences, University of Isfahan, Isfahan, Iran; 3 Research Institute of Biotechnology, Shahrekord University, Shahrekord, Iran; 4 Department of Biology, Faculty of Basic Sciences, Azad Islamic University of Shahrekord, Shahrekord, Iran; 5 Department of Physiology, Faculty of Medical Sciences, Shahrekord University of Medical Sciences, Shahrekord, Iran; 6 Institute of Biotechnology, Shiraz University, Shiraz, Iran; 7 School of Molecular and Biomedical Science, The University of Adelaide, Adelaide, Australia; Baylor College of Medicine, United States of America

## Abstract

An attractive approach to replace the destroyed insulin-producing cells (IPCs) is the generation of functional β cells from stem cells. Embryonal carcinoma (EC) stem cells are pluripotent cells which can differentiate into all cell types. The present study was carried out to establish a simple nonselective inductive culture system for generation of IPCs from P19 EC cells by 1–2 weeks old mouse pancreas extract (MPE). Since, mouse pancreatic islets undergo further remodeling and maturation for 2–3 weeks after birth, we hypothesized that the mouse neonatal MPE contains essential factors to induce in vitro differentiation of pancreatic lineages. Pluripotency of P19 cells were first confirmed by expression analysis of stem cell markers, Oct3/4, Sox-2 and Nanog. In order to induce differentiation, the cells were cultured in a medium supplemented by different concentrations of MPE (50, 100, 200 and 300 µg/ml). The results showed that P19 cells could differentiate into IPCs and form dithizone-positive cell clusters. The generated P19-derived IPCs were immunoreactive to proinsulin, insulin and insulin receptor beta. The expression of pancreatic β cell genes including, PDX-1, INS1 and INS2 were also confirmed. The peak response at the 100 µg/ml MPE used for investigation of EP300 and CREB1 gene expression. When stimulated with glucose, these cells synthesized and secreted insulin. Network analysis of the key transcription factors (PDX-1, EP300, CREB1) during the generation of IPCs resulted in introduction of novel regulatory candidates such as MIR17, and VEZF1 transcription factors, as well as MORN1, DKFZp761P0212, and WAC proteins. Altogether, we demonstrated the possibility of generating IPCs from undifferentiated EC cells, with the characteristics of pancreatic β cells. The derivation of pancreatic cells from EC cells which are ES cell siblings would provide a valuable experimental tool in study of pancreatic development and function as well as rapid production of IPCs for transplantation.

## Introduction

Diabetes mellitus is one of the most common chronic diseases which directly affects millions of people [1]. Type 1 diabetes can be ameliorated by islet transplantation. The concept of transplanting pieces of pancreas in diabetic patients has over a century history. However, the major problem is the deficiency of transplantable cadaver islets. Many studies have been focused on how to develop renewable sources of islet-replacement tissue. Whereas some studies have shown the generation of insulin-producing cells (IPCs) from progenitor cells of the pancreas [2], liver [3], [4], pluripotent embryonic stem (ES) cells [5]–[9], and skin-derived stem cells [10], the efficiency of in vitro generated IPCs is low. The existing protocols for generating IPCs from ES cells can be divided into spontaneous and induced differentiation [11]. In the present work, using neonatal mouse pancreas extract (MPE) as a natural biological inducer, we developed a simple accessible way to generate functional IPCs from P19 embryonal carcinoma (EC) stem cell line.

Total removal of the pancreas in dogs produces severe and fatal diabetes [12]. Daily injections of pancreatic extract prolonged life of a completely diabetic dog. Subcutaneous administration of whole pancreas extract to the human subjects caused decrease in blood sugar and increased utilization of carbohydrate [12]. Experimental studies demonstrated that the supplementation of oral nutrition with pancreatic extract-enriched diet improved the nutritional status of the aged rats [13], [14]. Reddy et al. showed that the oral administration of whole pancreas extract to young non-obese diabetic (NOD) mice, prevented autoimmune diabetes [15]. Rat pancreatic extract (RPE) enhanced the expression of the required transcription factors for pancreas development [16]. In vitro differentiation induction of rat mesenchymal cells into IPCs increased with the treatment of the cells by RPE [17], [18]. Using RPE as a natural biological inducer, Zhang et al. differentiated human amniotic mesenchymal stem cells into insulin-secreting cells [19]. Some studies show that RPE contains various growth factors and hormones related to pancreas regeneration [20], [21]. The effects of RPE on IPCs differentiation of human adipose tissue-derived stem cells (hASCs) were evaluated. Genes involved in early pancreas development (such as Sox17 and IPF-1) were expressed in RPE-treated culture [20].

Information regarding gene co-expression is useful to predict gene function [22], [23]. Several databases have been developed for gene co-expression analysis based on a large amount of publicly available gene expression data measured by GeneChip platforms. DNA microarrays deposited in public databases provide information on relative expression levels for thousands of genes simultaneously. In addition, large collections of microarray data contain information about concerted changes in transcript levels in these datasets beyond the original purpose of each dataset. Using the pattern of gene expression changes between two genes of interest in these concerted transcripts leads to similarity of expression (also called as “gene co-expression”). The hypothesis is that, if 2 genes show high correlation in different transcriptomics experiments/coditions, they are highly correlated to each other and is expected to show this high correlation in a specific phenomenon. Commonly, Pearson's correlation coefficient is used as a measure of gene co-expression. “1” indicates strong relationship in an aspect of gene expression regulation, “0” indicates no relationship. In this study, we employed a more recent expression measure called Mutual Ranking (MR) based on geometric mean of gene co-expression.

To provide an alternative source of IPCs for injection treatments, we developed a simple culture system using EC cell line and neonatal mouse pancreas extract and evaluate the differentiation of P19 cells into pancreatic β cells in the present investigation. P19 cells efficiently differentiated into IPCs and formed pancreatic islet-like structures. The P19-derived IPCs were immunoreactive to proinsulin, insulin and insulin receptor beta and could synthesize and secret insulin in response to glucose challenge. The P19-derived IPCs were also examined for the expression of the transcription factors which are required for pancreas development (PDX-1, EP300, CREB1). Network analysis of these key transcription factors during generation of the IPCs carried out based on co-expression analysis. Furthermore, to achieve a more comprehensive view on regulatory mechanisms governing the generation of IPCs, regulatory networks were designed by incorporating co-expression analysis results with the other types of interactions including microRNA inhibitory effect, promoter binding, and protein modification.

## Materials and Methods

### Ethics Statement

BALB/c pregnant mice were obtained from Azad University of Shahrekord (Shahrekord, Iran) and kept under standard housing conditions. All animal studies were approved by the ethical committee of Medical Sciences School from Azad University of Shahrekord, based on the National Specific Ethical Guidelines for Biomedical Research issued by Ministry of Health and Medicinal Education (MOHME) of Iran in 2005. Neonatal mice were anesthetized and then humanely sacrificed using cervical dislocation. All efforts were made to ameliorate suffering in the mice.

### Preparation of MPE

Whole pancreas of 1–2 weeks old newborn mice carefully removed and homogenized in PBS including protease inhibitors (PMSF, Roche, 10 837 091 001). Homogenates were centrifuged at 3000 rpm for 10 min at 4°C and then at 12,000 rpm for 20 min, to obtain the final clear supernatant. Immediately after obtaining the extracts, the samples were taken for analyzing the protein content using Bradford method. Extract was then stored as aliquots at −70°C until further use.

### Cell culture and differentiation induction protocol

Undifferentiated P19 cells (Pasteur Institute of Iran) were grown in α-MEM (Minimum Essential Medium, Gibco-BRL, Carlsbad, CA, 11900073) supplemented with 15% fetal bovine serum (FBS, Gibco, 10270-106), penicillin (50 µg/ml, Sigma, P3032), and streptomycin (50 µg/ml, Sigma, S1277) (stage 1). To direct the differentiation, the cells were grown for 4 days in suspension to induce embryoid body (EB) formation (stage 2). The EBs were cultured on 0.1% gelatin-coated petri dishes containing cover slip in α-MEM supplemented with 3% FBS for an additional 4 days and then cultured based on either spontaneous or induced differentiation protocol (stage 3). In order to induce the differentiation of the cells into IPCs, different concentrations of MPE (50, 100, 200, and 300 µg/ml) were added in stage 3. The cells cultured in medium with no MPE were considered as control.

### Dithizone (DTZ) staining

Dithizone staining was carried out as previously described [24] to assess quickly the presence of EC-derived IPCs. Fifty mg of DTZ (diphenylthiocarbozone, Merck, DX2370-3) in 5 mL of dimethylsulfoxide (DMSO, Sigma, D2650) was prepared as stock solution and stored at −20°C. In vitro DTZ staining was performed by adding 10 µL of the stock solution to 1 mL of culture medium, and then incubating at 37°C for 15–30 min. After three times rinsing, the dishes with HBSS (hank's balanced salt solution, sodium chloride 8000 mg/l, potassium chloride 400 mg/l, potassium phosphate monobasic KH_2_PO_4_ 60 mg/l, glucose 1000 mg/l, sodium phosphate dibasic Na_2_HPO_4_ anhyd. 48 mg/l), crimson red stained clusters were examined using a stereomicroscope. The number of stained cells in the cultures was counted using a hemocytometer under a microscope using an ×40 magnification. The assay was repeated at least five times.

### Immunoflourescence

The following primary antibodies were used in this study: mouse monoclonal proinsulin+insulin, (Abcam, ab8304-50) and mouse monoclonal insulin receptor beta (Abcam, ab8304-100). FITC-conjugated anti-mouse IgG (Sigma, St. Louis, MO, F9137) was used as a secondary antibody. Cells were cultured in 6-well plates on a cover slip, fixed in 4% paraformaldehyde, and permeabilized with 0.3% Triton X-100 in PBS for 30 min at 37°C. Following three washes with PBS, the cells were incubated with 10% normal goat serum (Sigma, G9023) in PBS for 30 min. Afterwards, the cells were incubated with the relevant primary antibody for 60 min, followed by incubation with the secondary antibody for 30 min. Cover slips were mounted with 70% glycerol in PBS. Several controls for immunostaining were used, and the primary antibody was omitted.

### Insulin detection assay by enzyme-linked immunosorbent assay (ELISA)

IPCs, undifferentiated P19 EC cells, and spontaneous differentiated EBs were grown in 6-well plates to estimate the intracellular and secreted insulin levels by ELISA. Before examination, the cells were washed three times with PBS and incubated in fresh serum-free medium with 0.5% bovine serum albumin (BSA) to enable the detection of insulin secretion without interference from the fetal serum. High-glucose challenge of the cells was achieved by the addition of serum-free media containing low (5.5 mmol/L)- or high (25 mmol/L)-glucose for 2 hours at 37°C. The conditioned media was collected and frozen at −70°C until assayed for insulin content. For measurement of the intracellular insulin content, cell pellets were sonicated in acid-ethanol (0.1 N hydrochloric acid in absolute ethanol). The values obtained were normalized in relation to the total protein content (protein assay reagent, Bradford, Sigma, B6916). ELISA was performed on the conditioned media and cellular extract using insulin mouse ultrasensitive ELISA kit (Alpco, 80-insmsu-E01).

### RNA extraction and real-time RT-polymerase chain reaction

Total cellular RNA was extracted from IPCs, undifferentiated P19 EC cells, and spontaneous differentiated EBs using Qiazol lyses reagent (Qiagene, 79306). Quantiscript reverse transcriptase (Qiagene -QuantiTect Rev_Transcription Kit, 205311) was used for synthesis of cDNA with oligo-dT and random primers, according to the manufacturer's protocol. RT-PCR assays were performed using the Qiagen apparatus (Qiagen, Rotor-Gene, California, USA). Real-time RT-PCRs of undifferentiated stem cell markers, Oct3/4 (POU Class 5 Homeobox 1), Sox-2 [SRY (Sex Determining Region Y)-Box 2] and Nanog (Nanog Homeobox) [25] as well as pancreatic β cell genes, PDX-1 (pancreatic and duodenal homeobox 1), INS1 (insulin1), INS2 (insulin2), EP300 (E1A binding protein p300), CREB1 (cAMP responsive element binding protein 1) and β-2M (β2 microglobulin) cDNAs were performed by using specific primers (Table 1) and SYBR Premix Ex Taq (TaKaRa, RR081Q). RT samples and negative controls (no template) were run together with test samples, and standard curves were used for each gene tested to analyze the efficiency of the PCR reaction. A melt curve analysis was performed at the end of each reaction. Expression levels were normalized to individual β-2M (internal control). The profile was obtained by plotting relative gene expression levels comparing to spontaneous differentiated EBs.

**Table 1:** Real time PCR Primer Sequences.

Gene	Primer sequences
PDX-1	F: 5′- TCCACCACCACCTTCCAGCTCA -3′R: 5′- TTCCTCGGGTTCCGCTGTGT -3′
INS1	F: 5′- ATGGCCCTGTTGGTGCACTTCC -3′R: 5′- AAGAAGCCACGCTCCCCACA -3′
INS2	F: 5′- AACATGGCCCTGTGGATGCG -3′ R: 5′- ACCCAGCTCCAGTTGTGCCA -3′
EP300	F: 5′- TCCATACCGAACAAAGGCCC -3′ R: 5′- CCAGATGTGCCCTGTTGTGT-3′
CREB1	F: 5′- AGAAGCAGCACGGAAGAGAG -3′R: 5′- CTTTCTGGTTGTGGCCAAGC -3′
Oct3/4	F: 5′- CTACCATCTGCCGCTTTG-3′R: 5′- GCCGCAGCTTACACATGTTCT-3′
Sox-2	F: 5′- ACAGCAAATGACAGCTGCAAA-3′R: 5′- TCGGCATCGCGGTTTTT-3′
Nanog	F: 5′- CCAAAGGCAAACAACCCACTT-3′R: 5′- CGGGACCTTGTCTTCCTTTTT-3′
β-2M	F: 5′- AGTCGTCAGCATGGCTCGCT -3′R: 5′- TGAGGCGGGTGGAACTGTGT -3′

### Statistical analysis

All experiments were repeated at least three times, and statistical significances were measured using Student's t-test, when there were two groups to be compared and one-way analysis of variance (ANOVA) and Duncan's Test, when there were more than two groups. Results were expressed as the mean ± standard deviation (SD) and P<0.05 was considered to be statistically significant.

### Co-expression analysis and co-expression-based network prediction of key IPC-producing TFs (PDX-1, EP300, and CREB1)

We used recent mutual ranking (MR) index instead of Pearson correlation coefficient as a co-expression measure by taking a geometric average of the Pearson correlation coefficient rank from gene A to gene B and that of gene B to gene A. Obayashi and Kinoshita, [26] documented that geometric average is a more accurate mature rather than arithmetic average since the differences of Pearson correlation coefficient ranks change as logarithmic manner [26]. In particular, the higher effectiveness of MR comparing to Pearson correlation coefficient is documented in large-scale microarray data of different organisms [26].

To perform co-expression analysis, deposited microarray data in GEO repository of NCBI were used via COXPRESSdb navigator (http://coxpresdb.jp/) [23]. We also analyzed the list of 100 co-expressed genes with each of PDX-1, EP300, and CREB1 TFs to find possible highly co-expressed genes with these TFs. Based on calculated MRs as a measure of co-expression/relation, co-expression-based network of PDX-1, EP300, and CREB1 were constructed.

### Regulatory-based (microRNA, literature, promoter binding) network of PDX-1, EP300, and CREB1

Besides co-expression, genes/proteins can interact to each other via promoter bonding, microRNA effect, protein modification, domain-domain interaction to build the network. These types of relations are the major causes of positive/negative correlation, signaling and regulatory effects. Using the found co-expressed genes of previous section as input genes, here, we built and visualize regulatory networks considering based on mirBase prediction database and literature mining by Pathway Studio 9 package (Elsevier) as previously described [27]. Statistically significant subnertworks were also identified by Fisher exact test implemented in Pathway Studio 9 package [28].

## Results

### Characterization of P19 EC cells on the basis of cell morphology and expression of pluripotent markers

**Figure 1:**
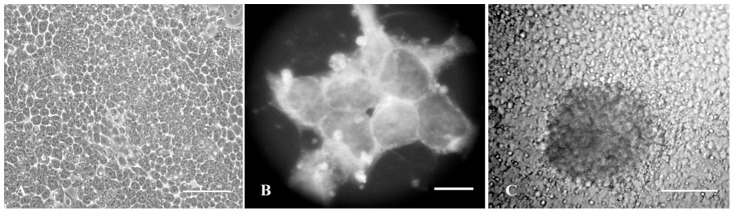
Representative micrographs showing morphology of exponentially growing P19 cells at low (A) and high (B) magnification. Undifferentiated cells are tightly packed polygonal cells, with large nucleoli and high nucleus–cytoplasm ratio. An embroyid body replated on 0.1% gelatin coated petri dish (C) differentiated spontaneously or induced by MPE. The cells spread out from the attached EB. Scale bars: A & C 20 µm, B 10 µm.

The experiments reported here were carried out with the P19 line of murine EC stem cells. At first, the characterization of the cells was carried out on the basis of cell morphology and expression of pluripotent markers. Undifferentiated P19 cells were polygonal in shape. Figure 1 shows the morphology of exponentially growing P19 cells at low (A) and high (B) magnification. When cultured in gelatinized dishes, the cells formed colonies composed of tightly packed polygonal cells of small size and prominent nuclei as presented in Figure 1. In non-adherent culture, P19 cells formed large spherical cell aggregates known as EBs. It is in accordance with the expected features of mouse and human ES cells [29], [30]. The EBs were then cultured on 0.1% gelatin-coated petri dishes containing cover slip. Figure 1 C shows an attached EB with the cells spread out from it. We also preformed Real-time PCR for assessing the expression of undifferentiated marker genes, including Oct3/4, Sox-2 and Nanog. The results showed that the expression of the genes was positive in P19 cells and significantly down regulated in differentiated cells (by 23.5, 9, and 4 times, respectively) (see below).

### Morphological studies of differentiated cells

**Figure 2:**
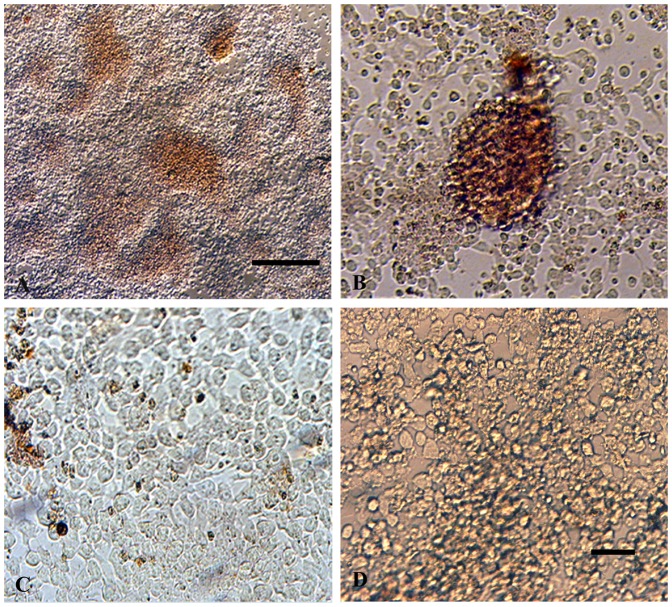
DTZ staining of differentiated IPCs, derived from P19 EC cells. IPCs formed pancreatic islet-like structures (A). A cell cluster distinctly stained crimson red by DTZ is apparent (B). Individual cells are DTZ-positive in spontaneous differentiated EBs (C). Untreated EC cells are not stained (D). Scale bars: A 100 µm, B, C & D 200 µm.

DTZ was used to quickly assess the presence of EC-derived IPCs. After DTZ staining, IPCs were identified as crimson red cell clusters. Figure 2 shows the results of DTZ-staining of P19-derived IPCs (Figure 2 A & B). Distinct DTZ-positive (DTZ^+^) cell clusters were observed in MPE-treated P19-derived EB outgrowths, while they were rare in spontaneous differentiated EBs (Figure 2 C) and obscure in the intact undifferentiated P19 cultures (Figure 2 D).

To estimate the frequency of the emerged DTZ^+^ cells in the cultures, the number of crimson red cells was directly counted under a microscope. Table 2 shows the percentage of DTZ^+^ cells among total cells in different concentrations of MPE. In all MPE-treated groups, the percentages of DTZ^+^ cells among total cells were significantly higher than untreated group. There was also significant difference between 50-, 100- and 200- and 300- µg/ml MPE-treated groups (P<0.05).

**Table 2:** Percentage of DTZ^+^ cells within total cells in different concentrations of mouse pancreas extract.

MPE (µg/ml)	DTZ^+^ (%) (mean±SD)
0	6.87±2.4^e^
50	25.57±5.72^d^
100	32.89±2.44^c^
200	37.22±3.76^b^
300	41.34±4.72^a^

The data present the means ± standard deviation (SD) of the means of DTZ^+^ cells derived from P19 EC cells in different concentrations of MPE used in the study. MPE induced the differentiation of P19 EC cells at a higher mean than that of the untreated group (0 µg/ml MPE: negative control). MPE: mouse pancreas extract, DTZ^+^: DTZ-positive cells.

### Detection of pancreatic β cell specific proteins

**Figure 3:**
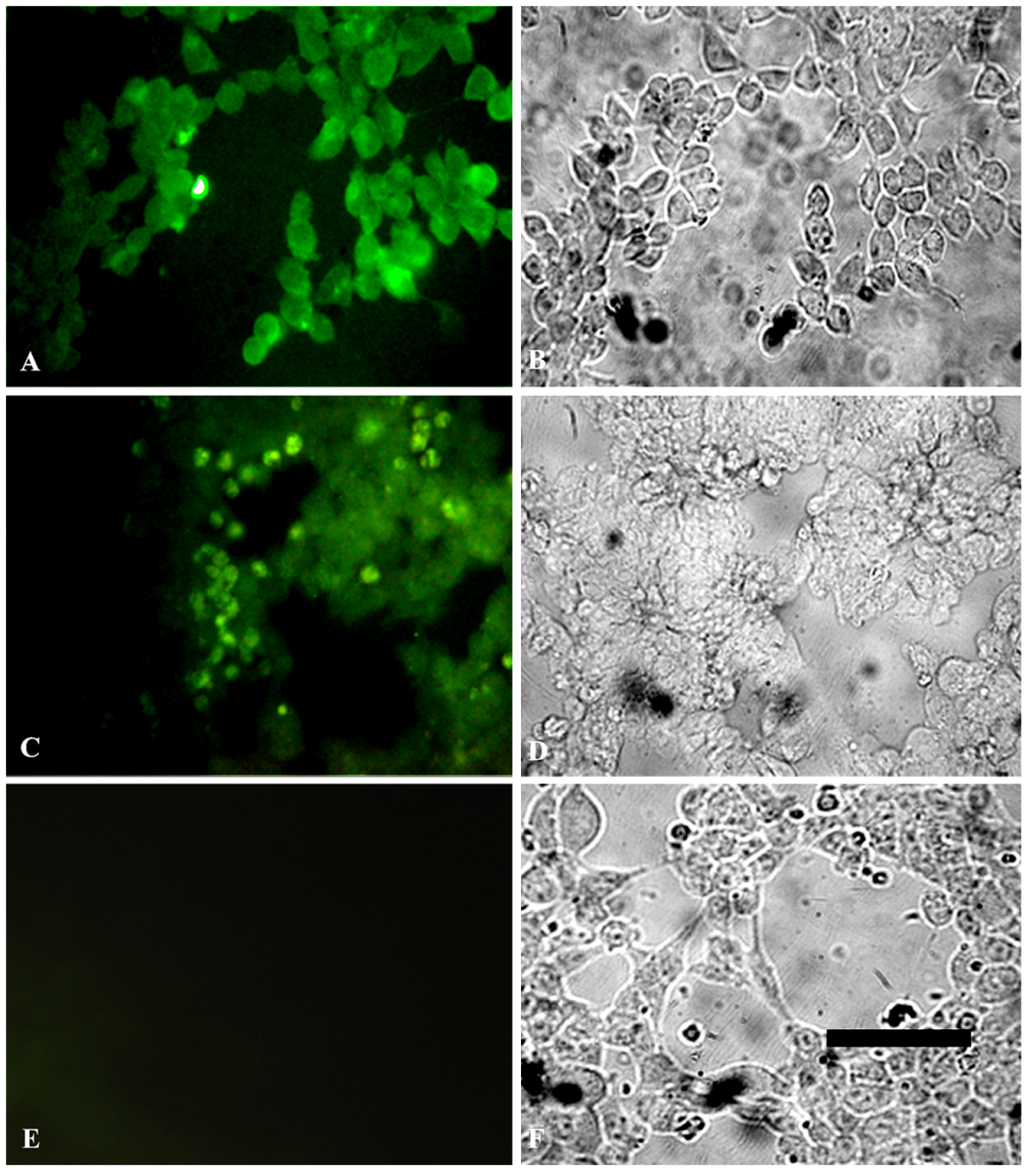
Fluorescence micrographs illustrating the expression of pancreatic β cell markers. Staining of IPCs with antibodies against proinsulin+insulin (A) and insulin receptor beta (C) showing that most of the P19 cells treated with MPE express pancreatic β cell markers. (E) Control for immunostaining, the primary antibody was omitted. B, D & F are phase contrast images of the same field shown in A, C & E respectively. Scale bars: 40 µm.

Immunoflourescence evaluation of the differentiated P19 cells showed that after MPE treatment these cells were immunoreactive to proinsulin, insulin (Figure 3 A) and insulin receptor beta (Figure 3 C). Several controls for immunostaining were used, and the primary antibody was omitted (Figure 3 E). The same fields were photographed as phase contrast images (Figure 3 B, D & F).

### Insulin secretion and content

**Figure 4:**
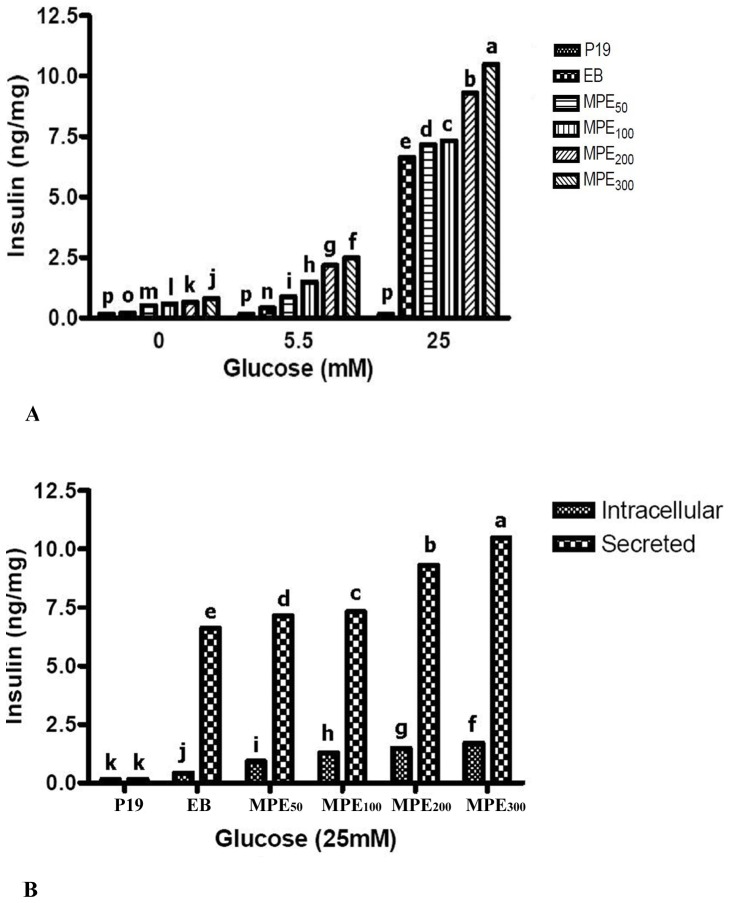
Determination of secreted (A) and secreted versus intracellular (B) insulin in P19 undifferentiated EC cells (P19), spontaneous differentiated EBs (EB) and the MPE-treated cells. Significant insulin concentration was observed in MPE–treated IPCs. To normalize the amount of insulin secretion, the total protein of the cells in each well was measured by the Bradford method. The experiment was performed in triplicate. Each value represents mean ± SD.

Glucose inducible insulin secretion was examined in P19 undifferentiated EC cells, spontaneous differentiated EBs and IPCs (Figure 4). The statistical differences between the groups used in the study revealed that compared to P19 and EB, differentiated IPCs demonstrated increase in secreted (Figure 4 A) and intracellular insulin (Figure 4 B), when normalized to the total protein content. Furthermore, when stimulated with glucose, IPCs synthesized and secreted insulin in a glucose-regulated manner as presented in Figure 4. Glucose-dependent insulin secretion was not observed in undifferentiated cells. The insulin secretion and content significantly enhanced when the glucose concentration in the medium was increased. The results showed that MPE could induce dose-dependent P19 differentiation into IPCs.

### Quantitative analysis of gene expressions

**Figure 5:**
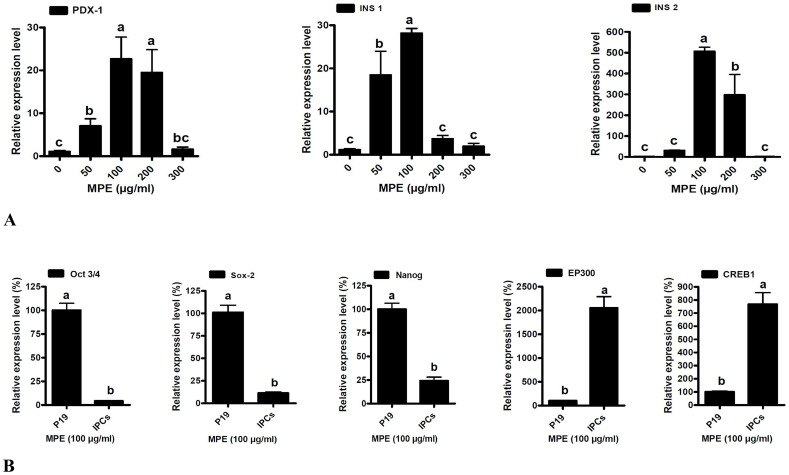
Quantitative analysis of genes involved in differentiation of pancreatic cells derived from P19 EC cells. The cells were cultured as EBs in different concentrations of MPE (mouse pancreas extract). (A) Relative gene expression of PDX-1, INS1, and INS2 in different concentration of MPE. (B) Comparison of expression of Oct3/4, Sox-2, Nanog (undifferentiated stem cell markers), and EP300, CREB1 (differentiated stem cell transcription factors) between P19 EC (embryonal carcinoma) cells and IPCs (insulin-producing cells). The data are expressed as relative gene expression to β-2M and are presented as mean±SD. The means with different letters are significantly different at P = 0.05.

Real-time qPCR analysis was used to examine the expression of pancreatic specific genes in differentiated cells. Results from representative experiments are shown in Figure 5. The data showed that in this culture system, transcription factors essential to pancreatic development such as PDX-1, INS1, INS2, EP300 and CREB1 were induced in the differentiated cells. The expression of PDX-1 was significantly increased in 50, 100 and 200 µg/ml concentrations of MPE compared with untreated EBs (Figure 5 A). There is no significant difference between 300 µg/ml of MPE and EBs in PDX-1 gene expression. MPE also significantly induced INS1 gene expression at concentration of 50 and 100 µg/ml (Figure 5 A). No significant differences existed between 200 and 300 µg/ml of MPE and control. Furthermore, the results showed that the INS2 gene expression was significantly higher at the concentrations of 100 and 200 µg/ml (Figure 5 A) in comparison with EBs. There was no significant difference between EB, 50 and 300 µg/ml. The expressions of these three genes were at the highest level in 100 µg/ml concentration of MPE compared with all the other groups.

In addition, to characterize the P19 EC cells, a real-time PCR analysis was performed on these cells and IPCs to compare undifferentiated marker genes, including Oct3/4, Sox-2 and Nanog and differentiated cell markers, EP300 and CREB1 (Figure 5 B). The results showed that the expression of Oct3/4, Sox-2 and Nanog is significantly down regulated in differentiated cells. EP300 and CREB1 were selected based on the predicted regulatory network. The expression of these transcription factors were more highly in IPCs than in P19 EC cells (Figure 5 B). During differentiation of IPCs from P19 EC, the expression of EP300 and CREB1 transcription factors was increased by 20 and 7.7 times, respectively. In contrast, a sharp decrease in expression of Oct3/4, Sox-2, and Nanog was observed by 23.5, 9, and 4 times, respectively (Figure 5 B).

### Co-expressed genes with PDX-1, EP300, and CREB1 transcription factors during IPC generation

Co-expressed genes with PDX-1, EP300, and CREB1 transcription factors were identified based on deposited microarray experiments in NCBI using COXPRESSdb navigator. The first 100 highly co-expressed genes with each of PDX-1, EP300, and CREB1 transcription factors and their Mutual Correlation Ranks (MR) are presented in Table S1, Table S2, and Table S3, respectively.

Interestingly, the two key TFs during IPC generation, EP300 and CREB1, had high level of co-expression in different microarray experiments where Mutual Correlation Rank was remarkably low (MR for Hsa ortholog = 5.1, Table S2). This guaranties highly significant co-expression and co-occurrence of EP300 and CREB1 during generation of IPC.

### Co-expression based network of PDX-1, EP300, and CREB1 transcription factors

**Figure 6:**
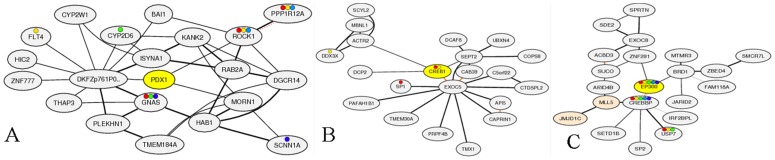
Co-expression based network of transcription factors involved in generation of insulin producing cells. (A) PDX1, (B) CREB1, and (C) EP300.

Based on co-expressed genes (selected by MR in different microarray experiments), expression networks were designed and visualized by COXPRESSdb (Figure 6). In addition, Functional annotations of these transcription factors were extracted based on Biological Process classification of Gene Ontology (Table S4).

As presented in Table S4, comparison of functional annotation of PDX-1, EP300, and CREB1 shows that PDX-1 has more glucose/insulin-dependent manner since PDX-1 is involved in GO groups of detection of glucose, pancreatic β cell differentiation, exocrine pancreas development, nitric oxide mediated signal transduction, and endocrine pancreas development. In contrast, EP300 and CREB1 are more involved in general differentiation and signaling functional classes such as N-terminal peptidyl-lysine acetylation, positive regulation of sarcomere organization, positive regulation of glycoprotein biosynthetic process, Toll signaling pathway, and positive regulation of transforming growth factor-beta3 production. The fact that EP300 and CREB1 are highly coregulated with each other and EP300 has binding sites on promoter regions of many genes reinforces the hypothesis that EP300 and CREB1 are shared background of differentiation and PDX-1 is a specific transcription factor for activation of IPCs employs. The union network consisting subnetworks activated by EP300 and CREB1 transcription factors is presented in Figure S1. The underpining significant subnetworks (p = 0.05) in EP300 CREB1 crosstalk such as neighbors of MAML2, LYL1, PPP2R5C, POU2F3, CRTC1, SLCO2A1, SIK2, TDG, PRKAR2B, VRK1, ATF5, and PLAGL1 are in listed Table S7. The relations and the corresponding references constructing EP300 CREB1 crosstalk is presented in Table S8.

PDX-1 has significant positive co-expression with MORN1, DKFZp761P0212, and ROCK1 (Table S4). ROCK1 protein kinase is a key regulator of actin cytoskeleton and cell polarity, can phosphorylate many proteins. However, DKFZp761P0212 is unknown protein and little information is available for MORN1. Consequently, MORN1 and DKFZp761P0212 are good targets for future studies regarding to IPCs generation.

### High co-expression of WAC and VEZF1 with both EP300, and CREB1 TFs: a potential shared subnetwork during differentiation

Comparing the commonly coregulated genes with PDX-1, EP300, and CREB1 transcription factors (Table S1, Table S2, and Table S3) in different microarray data showed that two genes WAC (domain containing adaptor with coiled-coil, Entrez Gene ID: 51322) and VEZF1 (vascular endothelial zinc finger 1, Entrez Gene ID: 7716) are shared between the first 100 co-expressed genes with both EP300, and CREB1 transcription factors.

WAC contains a WW domain, which is a protein module found in a wide range of signaling proteins; however, the exact function of WAC is not known. Coregulation of WAC, EP300, and CREB1 opens a new avenue in understanding WAC and VEZF1 transcription factor.

**Figure 7:**
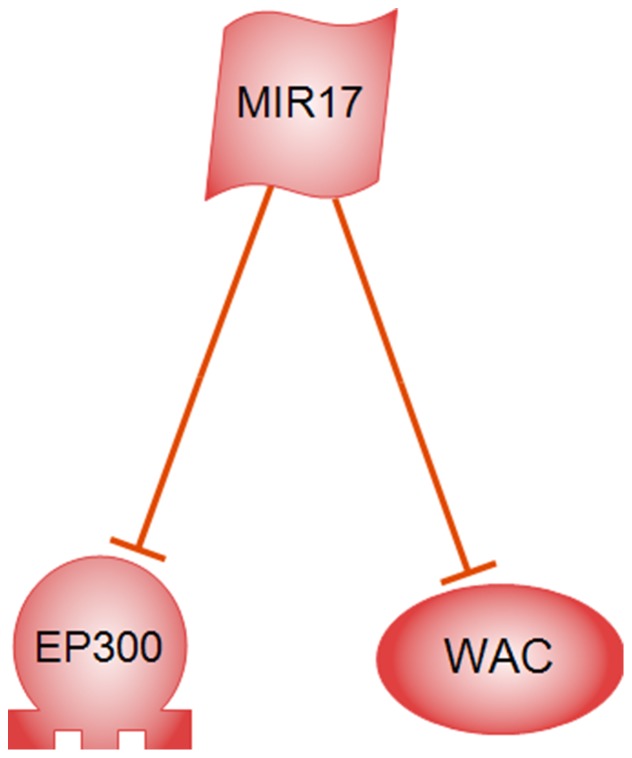
Crosstalk between highly co-expressed EP300 transcription factors and WAC protein based on MIR17 (microRNA17).

**Figure 8:**
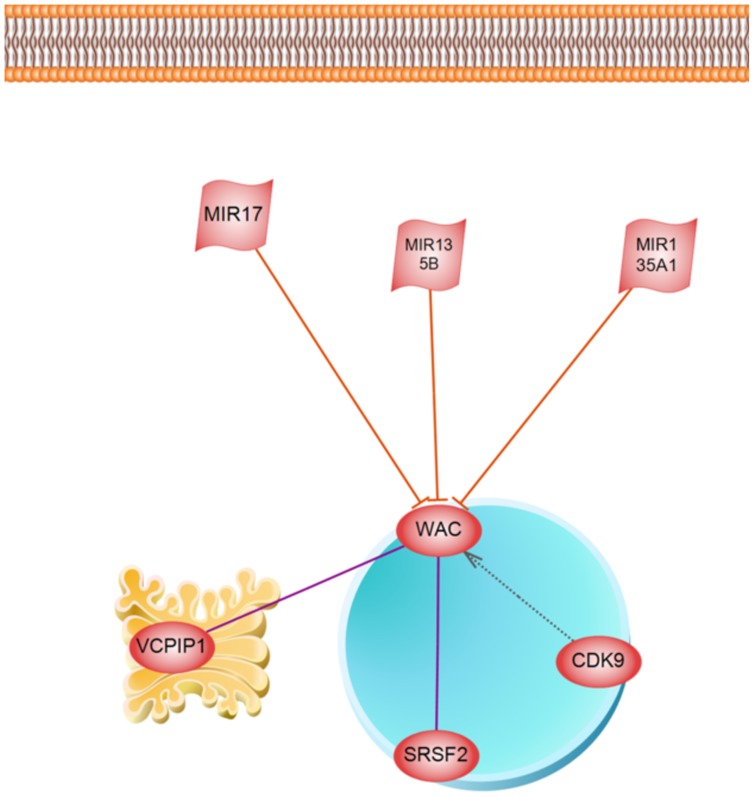
Regulatory network of WAC protein.

Supnetwork analysis of WAC, VEZF1, EP300, and CREB1 unraveled a new regulatory member, MIR17, which negatively regulates both WAC and EP300 (Figure 7). To get more clues on WAC function, a regulatory network was constructed based on literature mining and microRNA prediction which is presented at Figure 8. The underlying relations of this network are presented in Table S5. This network shows that WAC is under regulatory control of NIR17, MIR135B, and MIR135A1. WAC interacts with splicing factor SC35 (SRSF2) and CDK9 protein kinase.

**Figure 9:**
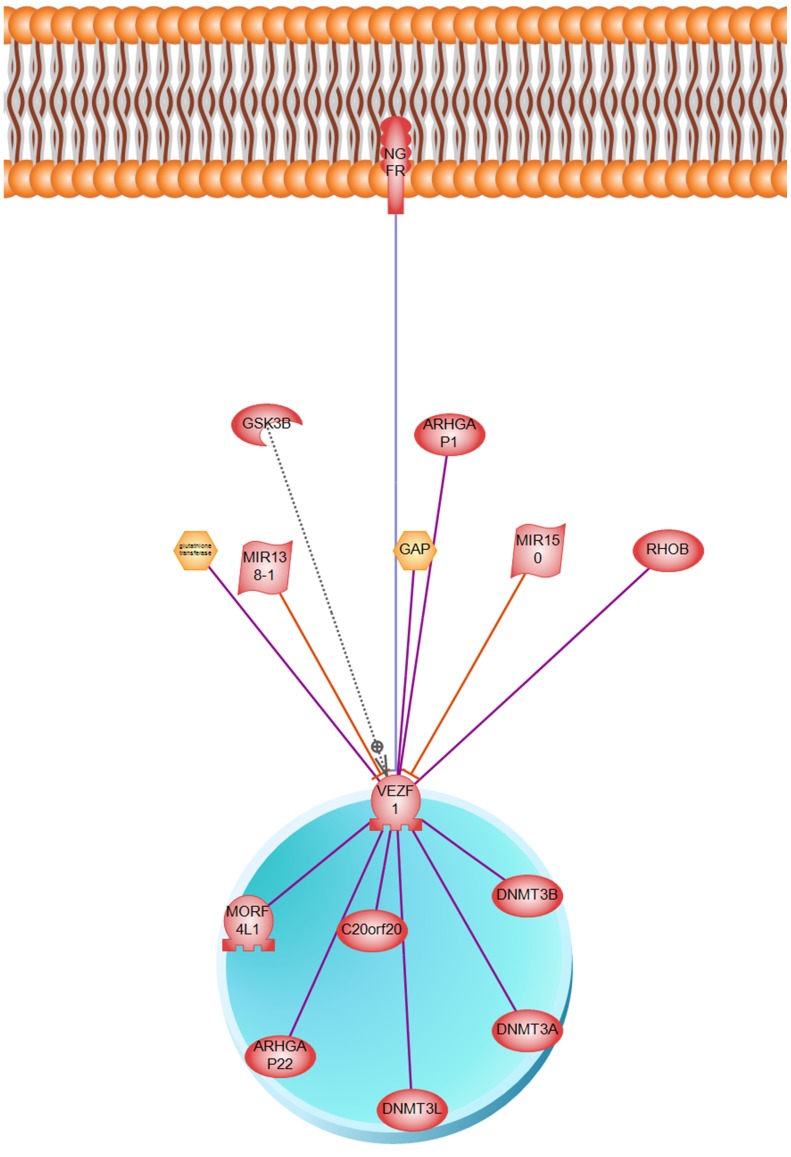
Regulatory network of VEZF1 protein.

VEZF1 is a transcriptional regulatory protein containing tandemly repeated zinc finger domains are thought to be involved in both normal and abnormal cellular proliferation and differentiation (http://www.genecards.org/). Designed regulatory network of VEZF1 transcription factor is presented in Figure 9 and its underpining relations and references are presented in Table S6. Architecture of VEZF1 transcription factor unravels an interesting signaling pathway transferring signal from nerve growth factor receptor (NGFR) located on cell membrane to nucleus. VEZF1 binds to the CT/GC-rich region of the interleukin-3 promoter and mediates tax transactivation of IL-3.

## Discussion

In the present study, we developed for the first time, a simple, accessible nonselective way to generate functional IPCs from P19 EC cell line within a short period of time (7–15 days). To induce IPCs differentiation, MPE from 1–2 weeks mouse neonate was used as a natural biological inducer. Our findings documented evidences that P19 cells could differentiate efficiently to functional IPCs by using MPE. It has been previously shown that pancreas extract contains various growth factors and hormones [20], [21]. Insulin-like growth factors (IGFs), insulin, transforming growth factor-b (TGF-b) and activin are expressed in the developing pancreas [31]–[33]. The exposure of stem cells to growth factors and extracellular matrix components may promote and streamline the differentiation process [11]. Since, mouse pancreatic islets undergo further remodeling and maturation for 2–3 weeks after birth [34], we hypothesized that the neonatal MPE contains essential factors to induce in vitro differentiation of pancreatic lineages. Previous studies showed that RPE which was obtained after pancreatectomy, could induce pancreas regeneration [16], [20], [21] and islet neogenesis in STZ-induced diabetic mice [35]. Moreover, RPE at a final concentration of 200 µg/ml, could stimulate differentiation of human adipose tissue-derived stem cells [20] and mesenchymal stem cells [17], [18]. Differentiation of human amniotic mesenchymal stem cells into IPCs by RPE has been also shown previously [19].

The existing protocols for generating IPCs from ES cells can be divided into spontaneous and induced differentiation [11]. The original protocols involve sequential in vitro differentiation steps to generate nestin expressing cells to derive IPCs from ES cells [36], [37]. However, Kahan et al. described a straightforward, nonsupplemented, nonselective protocol that supports the differentiation of mouse ES cells toward islet lineages. Their results indicated that under such spontaneous differentiation conditions, the cells were capable of differentiating into β like cells [38]. Here, we developed a simple nonselective inductive culture system rather than spontaneous differentiation of EC cells. It has been previously shown that the expansion of nestin-positive cells is not required for the activation of pancreatic differentiation [37]. Overall, our observations demonstrated that the differentiation of EC cells generated islet-like clusters, without selection of nestin-positive cells. A similar finding was documented by Blyszczuk et al. who induced ES cells differentiation into IPCs without induction and selection of nestin-expressing cells [39].

In accord with the earlier works, we demonstrated that MPE could induce pancreatic markers, proinsulin, insulin and insulin receptor beta. Expression of proinsulin is the main characteristic feature of normal pancreatic β cells [40]. Insulin receptor is a transmembrane receptor that is activated by insulin, IGF-I and IGF-II. These factors produced by pancreatic islet cells during development and tissue regeneration. They are strong stimulators of β cell replication and hypertrophy [41], [42]. Although the components of the MPE were not analyzed in this project, however, a possibility is that the soluble factors that exist in the extract were implicated in β cell differentiation via specific receptors expressed in IPCs.

Our observations documented that P19 cells could differentiate into functional β cells, capable of producing and secreting insulin in response to glucose. Both intracellular and secreted insulin enhanced when the glucose concentration in the medium was increased. Insulin secretion, especially under glucose induction, is an essential feature of pancreatic β cells [43]. It should be emphasized that immunoreactivity for both proinsulin and insulin in the current study indicated the presence of endogenously produced insulin. The observation is in line with previous findings of Fujikawa et al. who claimed that immunoreactivity for proinsulin or C-peptide indicates that the precursor proinsulin is synthesized by the differentiated cells [43]. Moreover, intracellular insulin level after exposure to 25 mM glucose was lower in comparison to secreted insulin (Figure 4 B). Therefore, it can be concluded that the hormone produced by the differentiated IPCs cells rather than taken up from the culture medium.

Real-time PCR analysis confirmed the expression of PDX-1 by the produced IPCs in our culture system. PDX-1 is a crucial regulator of pancreatic development [44] and its expression is very important in the in vitro differentiation of ES cells along pancreatic cell lineages [45]. Also, we found that mature two forms of insulin, INS1 and INS2 mRNA both expressed in the differentiated cells. These findings are in accordance with previous studies from our laboratory on IPCs differentiation of P19 cells by pancreas conditioned medium (submitted).

By comparing the expression of stem cell markers, we found Oct3/4, Sox2 and Nanog were more highly expressed in undifferentiated P19 cells than in IPCs. These transcription factors are markers for pluripotency and self-renewal in embryonic stem cells [46]. Oct3/4 is a POU domain-containing transcription factor highly expressed in ESCs [27]. Sox-2 is an HMG family protein which is required for maintenance of ESCs pluripotency similar to Oct4 [47]. A more recently described gene, Nanog, is thought to be a new “master gene” of ES cell pluripotency [48]. Functionally, Nanog blocks differentiation; thus, negative regulation of Nanog is required to promote differentiation during embryonic development [49]. Compared with P19 cells, the relatively reduced stem cell marker expression in IPCs may indicate their higher differentiation properties. Thus, it is possible that when EC cells were cultured as EBs, MPE could increase the level of specific pancreatic genes, while decreasing the expression of stem cell markers.

In this study, we used mutual rank (MR) instead of Pearson correlation coefficient since difference in gene expression correlation commonly follows a logarithmic manner rather than arithmetic average; consequently, geometric average (MR index) is more accurate as discussed by Obayashi and Kinoshita [26].

High correlation of EP300 and CREB1 in concert with WAC and VEZF1 transcriptomics data suggests common key regulators of differentiation. The fact that EP300 has high correlation with master transcription factors such as Sp2 transcription factor (Table S2) which has binding sites on the promoter regions of many genes reinforces this statement. On the other hand, PDX-1 can be seen as specific pancreas-development TFs which activates by inducers of pancreas development (MPE) and in concert with EP300, and CREB1 activates IPCs generation. The effect of MPE on PDX-1 is highly significant (p = 0.01) and MPE at 100 (µg/ml) is the best medium which results in the highest expression of PDX-1 according to Tukey test at p = 0.05 (data not shown).

## Conclusion

To best of our knowledge, this is the first report demonstrating the differentiation of P19 EC cells into IPCs in a simple and accessible way. By using MPE as a natural inducer, we successfully differentiated P19 cells into mature islet-like cell clusters with the molecular and functional characteristics of pancreatic β cells without selection of nestin-expressing cells. The derivation of pancreatic cells from EC cells which are ES cell siblings would provide a valuable experimental tool to study pancreatic development and function. This method may provide a new way to substitute the cytokines required in IPCs induction of stem cells. The current study resulted in new candidates in generation of IPCs including WAC, VEZF1, MIR17, MORN1, and DKFZp761P0212 for future studies. All together, bioinformatics approaches employed in this study helps in discovery of new elements of insulin-producing cell mechanism and gene discovery based on network construction.

## Supporting Information

Figure S1 **Significant subnetworks (Union selected subnetworks) in CREB1 EP300 crosstalk.**
(TIF)

Table S1 **Highly co-expressed genes with PDX1 transcription factor in different microarray experiments.**
(XLSX)

Table S2 **Highly co-expressed genes with EP300 transcription factor in different microarray experiments.**
(XLSX)

Table S3 **Highly co-expressed genes with CREB transcription factor in different microarray experiments.**
(XLSX)

Table S4 **Functional annotation and highly co-expressed genes with the key transcription factors (PDX-1, EP300, and CREB1) governing the generation of insulin-producing cells.**
(DOCX)

Table S5 **Relationships and references constructing WAC regulatory network (** **Figure 8** **).**
(XLSX)

Table S6 **Relationships and references constructing VEZF1 regulatory network (** **Figure 9** **).**
(XLSX)

Table S7 **Significant subnetworks in EP300 CREB crosstalk (Figure S1).**
(XLSX)

Table S8 **Relations and their references constructing CREB1 EP300 crosstalk network (Figure S1).**
(XLSX)
