# Supplementary figures and images for: Efficient and Simple Production of Insulin-Producing Cells from Embryonal Carcinoma Stem Cells Using Mouse Neonate Pancreas Extract, As a Natural Inducer
Source: PLoS One. 2014 Mar 10;9(3):e90885. doi: 10.1371/journal.pone.0090885 (PMC3948699; doi:10.1371/journal.pone.0090885)

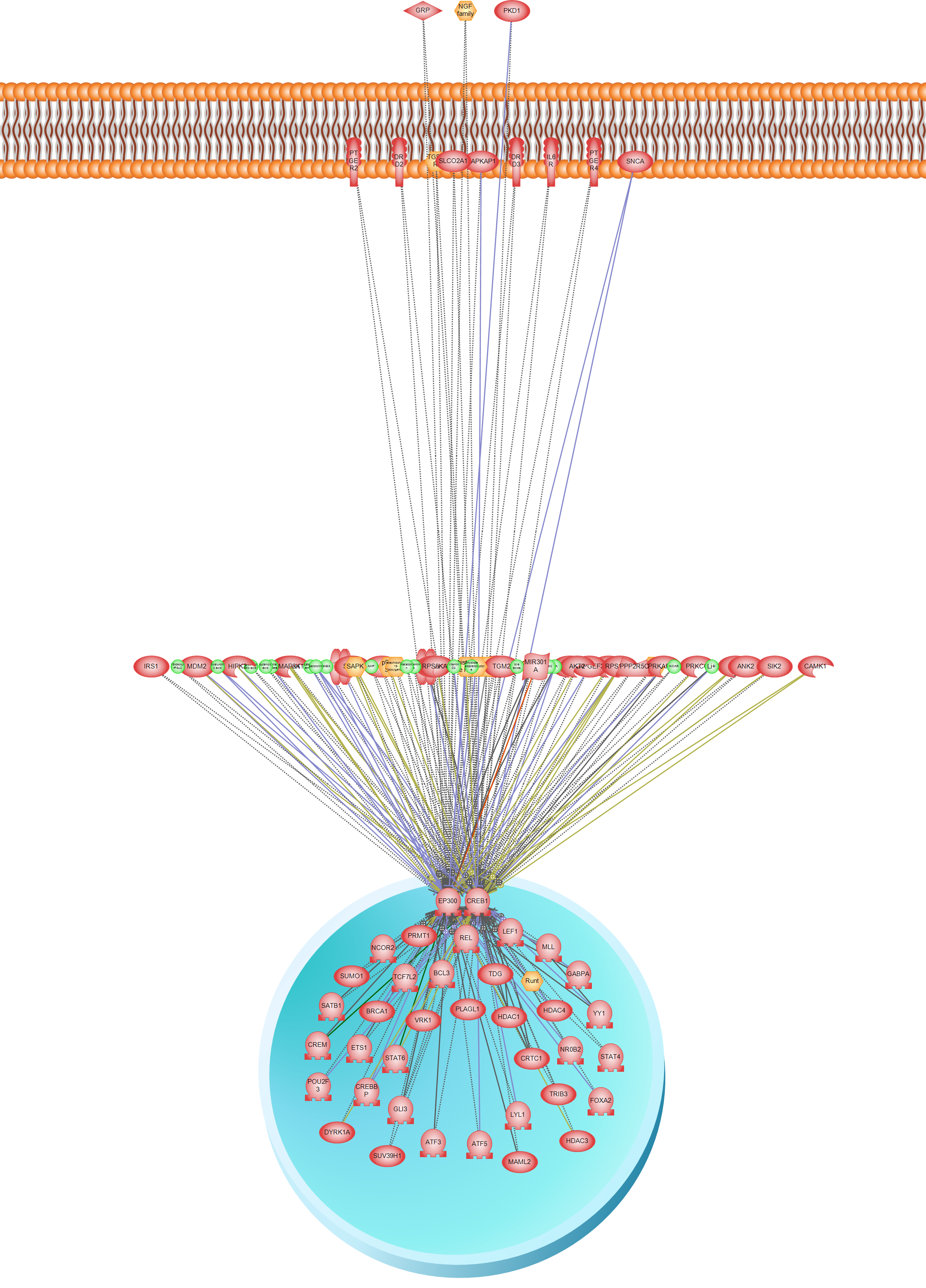

Supplement: Figure S1 — Significant subnetworks (Union selected subnetworks) in CREB1 EP300 crosstalk. (TIF) [file pone.0090885.s001.tif]
