# Supplementary material for: Efficient and Simple Production of Insulin-Producing Cells from Embryonal Carcinoma Stem Cells Using Mouse Neonate Pancreas Extract, As a Natural Inducer
Source: PLoS One. 2014 Mar 10;9(3):e90885. doi: 10.1371/journal.pone.0090885 (PMC3948699; doi:10.1371/journal.pone.0090885)
Supplement: Table S4 — Functional annotation and highly co-expressed genes with the key transcription factors (PDX-1, EP300, and CREB1) governing the generation of insulin-producing cells. (DOCX) [file pone.0090885.s005.docx]

**Supplementary 4**. Functional annotation and highly co-expressed genes with the key transcription factors (PDX-1, EP300, and CREB1) governing the generation of insulin-producing cells

| Transcription factor | functional annotation (biological process) | | |  | Transcription factor | functional annotation (biological process) | | |  | Transcription factor | functional annotation (biological process) | | |
| --- | --- | --- | --- | --- | --- | --- | --- | --- | --- | --- | --- | --- | --- |
| PDX-1 (pancreatic and duodenal homeobox 1) | GO:0051594 | detection of glucose | |  | EP300 (E1A binding protein p300) | GO:0018076 | N-terminal peptidyl-lysine acetylation | |  | CREB1  (cAMP responsive element binding protein 1) | GO:0008063 | Toll signaling pathway | |
|  | GO:0003309 | pancreatic B cell differentiation | |  |  | GO:0060298 | positive regulation of sarcomere organization | |  |  | GO:0002756 | MyD88-independent toll-like receptor signaling pathway | |
|  | GO:0031017 | exocrine pancreas development | |  |  | GO:0032025 | response to cobalt ion | |  |  | GO:0034138 | toll-like receptor 3 signaling pathway | |
|  | GO:0007263 | nitric oxide mediated signal transduction | |  |  | GO:0043923 | positive regulation by host of viral transcription | |  |  | GO:0032916 | positive regulation of transforming growth factor-beta3 production | |
|  | GO:0031018 | endocrine pancreas development | |  |  | GO:0010560 | positive regulation of glycoprotein biosynthetic process | |  |  | GO:0034134 | toll-like receptor 2 signaling pathway | |
|  |  |  | |  |  |  |  | |  |  |  |  | |
| The main coexpressed genes with PDX-1 in PDX-1 expression network | | | | | | | | | | | | | |
| symbol | fuction | | MR (Mutual Rank) with PDX-1 |  | Coexpression detail (microarray experiment) for PDX1 association | | | | | | | | |
| MORN1 | MORN repeat containing 1 | | 13.7 |  | GEO microarray experiment IDs: GSE5281, GSE2109, GSE6575, GSE6532, GSE4757, GSE5809, GSE4757, | | | | | | | | |
| DKFZp761P0212 | uncharacterized protein DKFZp761P0212 | | 22.5 |  | GEO microarray experiment IDs: GSE2109, GSE5281, GSE6575, GSE5281, GSE2634, GSE4757 | | | | | | | | |
| ROCK1 | Rho-associated, coiled-coil containing protein kinase 1 | | 25.0 |  | GEO microarray experiment IDs: GSE5281, GSE6575, GSE2109, GSE2109 | | | | | | | | |
|  |  | |  |  |  |  | |  |  |  |  | |  |
| The main coexpressed genes with EP300 1 in EP300 expression network | | | | | | | | | | | | | |
| symbol | fuction | | MR (Mutual Rank) with PDX-1 |  | Coexpression detail (microarray experiment) for EP300 association | | | | | | | | |
| CREBBP | CREB binding protein | | 5.1 |  | GEO microarray experiment IDs: GEO microarray experiment IDs: GSE2109, GSE7835, , GSE6969, GSE2109, GSE5281, GSE7213, GSE6400, GSE5809, GSE6400, GSE5787, GSE2634, | | | | | | | | |
| ZNF281 | zinc finger protein 281 | | 6.5 |  | GEO microarray experiment IDs: GSE7835, GSE5281, GSE2634, GSE6400, GSE2109, GSE6400, GSE6969, GSE6969, GSE2109, GSE2634 | | | | | | | | |
| BRD1 | bromodomain containing 1 | | 8 |  | GEO microarray experiment IDs: GSE7835, GSE5281, GSE2109, GSE6791 | | | | | | | | |
|  |  | |  |  |  |  | |  |  |  |  | |  |
| The main coexpressed genes with CREB1 in CREB1 expression network | | | | | | | | | | | | | |
| symbol | fuction | | MR (Mutual Rank) with PDX-1 |  | Coexpression detail (microarray experiment) for EP300 association | | | | | | | | |
| EXOC5 | exocyst complex component 5 | | 4.5 |  | GEO microarray experiment IDs: GSE3526, GSE5675, GSE2677, GSE3526, SE5675, GSE2677, GSE6338, GSE2109, GSE2677, GSE5350, GSE3744, GSE6013, | | | | | | | | |
| SEPT2 | septin 2 | | 6.0 |  | GEO microarray experiment IDs: GSE6338, GSE6338, GSE2109, GSE3744, GSE5350 | | | | | | | | |
| ACTR2 | ARP2 actin-related protein 2 homolog (yeast) | | 9.0 |  | GEO microarray experiment IDs: GSE2677, GSE2109, GSE7127, GSE5675, GSE6013, GSE4036 | | | | | | | | |
| DCP2 | DCP2 decapping enzyme homolog (S. cerevisiae) | | 13.2 |  | GEO microarray experiment IDs: GSM53147, GSM102443, GSM102505, , GSM88958, GSM155675, GSM80800, GSM117679, GSM38346, GSM46903, GSM119645 | | | | | | | | |
